# Mental Health Care Use Among Children and Adolescents With High Health Care Costs in Ontario, Canada

**DOI:** 10.1001/jamanetworkopen.2023.13172

**Published:** 2023-05-12

**Authors:** Claire de Oliveira, Tomisin Iwajomo, Paul Kurdyak

**Affiliations:** 1 Institute for Mental Health Policy Research and Campbell Family Mental Health Research Institute, Centre for Addiction and Mental Health, Toronto, Ontario, Canada; 2 ICES, Toronto, Ontario, Canada; 3 Institute of Health Policy, Management, and Evaluation, University of Toronto, Toronto, Ontario, Canada; 4 Department of Psychiatry, Faculty of Medicine, University of Toronto, Toronto, Ontario, Canada; 5 Mental Health and Addictions Centre of Excellence, Ontario Health, Toronto, Ontario, Canada

## Abstract

**Importance:**

Research on patients with high health care costs has examined mainly adults, with little focus on mental health care use.

**Objective:**

To examine the characteristics and costs of children and adolescents with high health care costs who use mostly mental health care and whether and why they persist in the high-cost state.

**Design, Setting, and Participants:**

This population-based, retrospective cohort study used health care records from Ontario, Canada, on all children and adolescents (age 0-17 years) covered under a universal health care system from January 1, 2012, to December 31, 2019. All children and adolescents in and above the 90th percentile of the cost distribution in 2012 for whom costs related to mental health care accounted for 50% or more of their costs were defined as patients with high mental health care costs. Data were analyzed from August 2019 to December 2022.

**Exposures:**

High health care costs.

**Main Outcomes and Measures:**

Patients with high mental health care costs were characterized in terms of their sociodemographic characteristics; chronic physical health, mental health, and behavioral conditions; and health care costs (in 2021 Canadian dollars) by health service and type of care (mental health care vs non–mental health care). Patients were followed up until 2019 to assess whether they persisted in the high-cost state and to examine factors associated with persisting in that state.

**Results:**

In 2012, there were 273 490 children and adolescents with high health care costs (mean [SD] age, 6.43 [5.99] years; 55.8% male; mean cost, $7936.40; 95% CI, $7850.30-$8022.40). Of these, 20 463 (7.5%) were classified as having high mental health care costs (mean cost, $10 040.20; 95% CI, $9822.80-$10 257.50). Asthma (30.3%), attention-deficit/hyperactivity disorder (35.8%), and mood and/or anxiety disorders (94.9%) were the most common chronic physical, behavioral, or mental health conditions. Few patients with high mental health care costs persisted in the high-cost state beyond 3 years (19.0%). Mood and/or anxiety disorders (relative risk ratio [RRR], 6.17; 95% CI, 3.19-11.96) and schizophrenia spectrum disorders (RRR, 2.98; 95% CI, 2.14-4.14) were identified as the main factors associated with persistence in the high-cost state.

**Conclusions and Relevance:**

In this cohort study of children and adolescents with high health care costs, some patients had high levels of mental health care use and high costs of care, but few of these persisted in the high-cost state for 3 or more years. These findings may help inform the development of care coordination interventions and service delivery models, such as youth integrated services, to reduce costs and improve outcomes for children and adolescents.

## Key Points

**Question:**

What are the characteristics and costs of children and adolescents with high health care costs who use mainly mental health care, and do they persist in the high-cost state?

**Findings:**

In this population-based cohort study of 273 490 Canadian children and adolescents, among those with high health care costs, some had high levels of mental health care use, namely those with mood and/or anxiety disorders. Few children and adolescents (19%) persisted in the high-cost state for 3 or more years, and most who did had mood and/or anxiety and schizophrenia spectrum disorders.

**Meaning:**

These findings may be helpful for developing appropriate case management and care coordination interventions and improving the design of care pathways for this population.

## Teaser

This cohort study examines the characteristics and costs of children and adolescents in Canada with high health care costs who use mostly mental health care, the proportion of patients with persistently highest costs, and the factors associated with persisting in a high-cost state.

## Introduction

Research on patients with high health care costs has typically focused on adults. However, despite having lower overall costs than adults,^1^ children and adolescents with high health care costs, particularly those with mental illness, are an important population to examine as many chronic conditions originate in childhood and adolescence and can lead to poor later-life outcomes.^2^ Research on high health care utilization and costs has mainly focused on children with medical complexity and lifelong physical conditions (eg, severe neurologic impairment), children requiring technology assistance, or premature neonates requiring complex and expensive hospital care.^3,4^ One study^3^ found that although accounting for less than 1% of the child population in Ontario, Canada, children with medical complexity accounted for one-third of all pediatric health care costs. In the US, researchers found that children with medical complexity accounted for 6% of all children with Medicaid coverage and 34% of all spending; the most prevalent chronic conditions were neurologic or neuromuscular (25%), congenital or genetic (22%), and cardiovascular (19%).^4^

Children and adolescents with mental illness are rarely identified as patients with high health care costs, partly because relatively few children with mental illness are admitted to the hospital^5^ but also because mental health care use is rarely measured in this patient population. Care for children with mental illness can fall under the responsibility of many different ministries or departments (eg, health, education, or youth services), making it challenging to measure mental health care costs, thus requiring a whole-system perspective. Among the existing work that has examined children and adolescents with high health care costs, studies have found that the most frequent diagnoses include mental^1,6,7,8^ and behavioral disorders^6,7^ and that children and adolescents with these diagnoses typically have 1 or more complex chronic conditions.^6,7^ However, existing work has mainly used nonrepresentative samples (eg, Medicaid,^4,7^ private insurance^6^), and few studies have examined mental health–related costs in detail.^6,7^ Furthermore, little work has studied persistence in the high-cost state^6^; understanding whether patients persist in this state is important to inform future interventions. Using health records of children and adolescents covered under a universal health care system, this study examined patients with high health care costs who used mostly mental health care and whether and why they persisted in the high-cost state.

## Methods

### Study Design and Setting

We undertook a retrospective, population-based, longitudinal cohort study of children and adolescents with high health care costs who were aged 0 to 17 years in 2012 to examine those who used mostly mental health care from January 1, 2012, to December 31, 2019, using data from Ontario, Canada’s most populous province (2019 population, 14.6 million). Most health care received by legal residents of Ontario eligible for public health insurance is provided under a universal, single-payer health care system funded through general taxation. We used health records available at ICES, an independent, nonprofit research institute in Toronto, Ontario. The use of these data was authorized under section 45 of Ontario’s Personal Health Information Protection Act, which does not require review by a research ethics board or patient informed consent. This study was conducted according to the Strengthening the Reporting of Observational Studies in Epidemiology (STROBE) reporting guideline.

### Data

The ICES data repository contains individual-level linkable and longitudinal data on most publicly funded health care services for all legal residents of Ontario. We used the following databases: Discharge Abstract Database; Ontario Mental Health Reporting System; Continuing Care Reporting System; National Rehabilitation Reporting System; National Ambulatory Care Reporting System; Ontario Health Insurance Plan claims database; Ontario Drug Benefit database; Home Care Database; Registered Persons Database (a population-based registry); Immigration, Refugees and Citizenship Canada database; and census data. A full description of each database can be found in eTable 1 in Supplement 1. All databases were linked using unique encoded identifiers, termed “ICES key numbers,” generated using a secure ICES algorithm based on an individual’s health card number and analyzed at ICES in compliance with Ontario privacy legislation.

### Population

We selected all individuals aged 0 to 17 years with a valid health card number and at least 1 encounter with the health care system in 2012 to obtain a comprehensive picture of all children and adolescents with health care costs. Among these, we defined children and adolescents with high costs as all those in and above the 90th percentile of the cost distribution, as done previously,^9,10,11^ obtaining a larger cohort of patients compared with other definitions (eg, 95th percentile).^1,8^ Children and adolescents with high mental health care costs were defined as those for whom costs related to mental health care use (eg, all psychiatric-related hospital and physician care, drugs, and home care) accounted for 50% or more of their total health care costs, in line with a previously developed algorithm.^9^ All other children and adolescents were defined as having high non–mental health care costs.

### Cost Estimation

All direct patient-level health care costs borne by the public third-party payer, the Ontario Ministries of Health and Long-Term Care, were estimated with a costing algorithm^12^ that used a bottom-up, microcosting approach to cost services at the patient level. Given Ontario’s public health insurance system, private marketplace providers rarely set prices; therefore, costs or amounts paid by the public third-party payer were used. When individual unit costs were unavailable (eg, long-term care), a top-down approach, which allocates aggregate costs to individual visits or individual cases or episodes of care, was used. Costs captured by the algorithm account for over 90% of all government-paid health care services.^12^ Further details on the costing method can be found elsewhere.^12^ Costs were categorized into several categories: psychiatric hospitalizations, acute medical hospitalizations, other hospital- or institution-based care (ie, complex continuing care, inpatient rehabilitation, and long-term care), hospital outpatient clinic visits, emergency department visits, other ambulatory care (ie, same-day surgery, dialysis clinic visits, and cancer clinic visits), physician services, outpatient prescription drugs covered under the public provincial drug plan, and home care. Costs are reported in 2021 Canadian dollars.

### Defining Children and Adolescents With Persistent High Costs

We observed patients with high mental health care costs only from 2012 to 2019 to examine whether they moved in and out of the high-cost state over time. Prior work has defined patients with persistent high costs as those who incur high costs consistently and continuously over several periods^13,14^; therefore, we defined children and adolescents with persistent high costs as those in the high-cost state for 6 or more years (at any point) over the 8-year period. We defined patients with sporadic and moderate high costs as those in the high-cost category for 1 to 2 years and 3 to 5 years, respectively. Some patients did not have data for all years due to loss to follow-up (eg, decedents, moved out of the province, or were no longer eligible for public health care insurance); these patients were defined as the attrition cohort. We classified high-cost status similarly in the attrition and nonattrition cohorts and included both in the descriptive analysis.

### Statistical Analysis

Children and adolescents with high costs were characterized in terms of mean and median costs, sociodemographic characteristics (sex, age, migrant status, neighborhood income quintile, rural residence, and administrative health region), and presence of chronic physical, mental, and behavioral conditions (ie, conditions typically regarded as being chronic) common among children and adolescents^4,6,7,8^ (asthma, cancer, cerebral palsy, congenital heart disease, Crohn disease or colitis, diabetes, epilepsy, spina bifida, attention-deficit/hyperactivity disorder [ADHD], mood and/or anxiety disorders, autism spectrum disorder, severe eating disorders, and schizophrenia spectrum disorders) ascertained either through disease registries (Pediatric Oncology Group of Ontario Networked Information System and Ontario Cancer Registry), validated algorithms, or other algorithms used elsewhere that use hospital-based records and existing physician billing codes.^15,16,17,18,19,20,21,22,23,24,25,26,27^ Patients with high mental and nonmental health care costs were then compared. We also estimated mean costs and respective 95% CIs by care and services related to mental and nonmental health for each group in 2012.

Next, we compared sociodemographic characteristics and presence of chronic conditions among patients with sporadic, moderate, and persistent high mental health care costs only and estimated total mean health care costs for each subgroup from 2012 to 2019. Finally, we used a multinomial logistic regression model^28,29^ on the pooled data (ie, all 8 years of data combined) to examine persistence in the high-cost state and assess factors associated with being a child or adolescent with sporadic vs moderate or persistent high mental health care costs, in which the dependent variable for each patient was constructed using the aforementioned definitions. This model controlled for sociodemographic characteristics, chronic conditions, administrative health region, and calendar year, which could vary over time. The model coefficients were exponentiated to obtain the relative risk ratio (RRR) for a 1-unit change in the corresponding variable (in which risk was measured as the risk of the outcome compared with the base outcome [ie, sporadic status]).^28,29^ Clustered SEs were estimated for all models to account for repeated observations for the same individual when applicable.

We undertook a sensitivity analysis in which attritors were excluded to examine the robustness of the findings. All analyses were performed using SAS Enterprise Guide, version 7.1 (SAS Institute Inc). Data were analyzed from August 2019 to December 2022.

## Results

In 2012, 273 490 children and adolescents were classified as patients with high health care costs, with a mean cost of $7936.40 (95% CI, $7850.30-$8022.40) (Table 1). Of these individuals, 44.2% were female and 55.8% were male, and the mean (SD) age was 6.43 (5.99) years; 41.7% were aged 0 to 3 years. The majority were long-term residents (96.6%) and had an urban residence (88.8%), and the distribution among neighborhood-level income quintiles was roughly equal. The most common chronic conditions were asthma (22.9%) and mood and/or anxiety disorders (30.1%). Many patients with high costs (48.4%), mostly newborns, did not have any chronic conditions. Findings were somewhat similar at the 95th percentile, at which individuals did not differ on sociodemographic characteristics but had slightly more comorbidities than those in the 90th percentile; findings differed at the 99th percentile, as individuals had more physical comorbidities (but fewer mental comorbidities) than in the other 2 groups. Costs in the overall group with high health care costs were mainly due to acute medical hospitalizations (39.8%) and physician care (21.6%) (Table 2).

**Table 1.** Characteristics of Children and Adolescents With High Health Care Costs in Ontario, Canada, 2012

Characteristic	Patients with high costs^a^		
	Full sample (N = 273 490)	Mental health care (n = 20 463)	Non–mental health care (n = 253 027)
**Cost^b^**			
Mean (95% CI)	7936.40 (7850.30-8022.40)	10 040.20 (9822.80-10 257.50)	7766.20 (7674.90- 7857.50)
Median (IQR)	3611 (2643-6190)	4423 (2845-11 650)	3568 (2631-5999)
**Sociodemographic characteristics**			
Sex			
Female	120 853 (44.2)	9953 (48.6)	110 900 (43.8)
Male	152 637 (55.8)	10 510 (51.4)	142 127 (56.2)
Age, y			
Mean (SD)	6.43 (5.99)	12.84 (3.81)	5.92 (5.83)
Median (IQR)	5 (0-12)	14 (11-16)	5 (0-11)
0-3	113 959 (41.7)	588 (2.9)	113 371 (44.8)
4-8	61 073 (22.3)	2573 (12.6)	58 500 (23.1)
9-13	44 549 (16.3)	5879 (28.7)	38 670 (15.3)
14-17	53 909 (19.7)	11 423 (55.8)	42 486 (16.8)
Migrant status			
Canadian born or long-term resident	264 062 (96.6)	19 407 (94.8)	244 655 (96.7)
Immigrant	7376 (2.7)	860 (4.2)	6516 (2.6)
Refugee	2052 (0.8)	196 (1.0)	1856 (0.7)
Neighborhood income quintile^c^			
Missing	1485 (0.6)	138 (0.7)	1347 (0.5)
1	56 931 (20.8)	3856 (18.8)	53 075 (21.0)
2	51 421 (18.8)	3586 (17.5)	47 835 (18.9)
3	53 719 (19.6)	3907 (19.1)	49 812 (19.7)
4	58 750 (21.5)	4416 (21.6)	54 334 (21.5)
5	51 184 (18.7)	4560 (22.3)	46 624 (18.4)
Rural residence			
Missing	207 (0.1)	61 (0.3)	146 (0.1)
No	242 943 (88.8)	18 582 (90.8)	224 361 (88.7)
Yes	30 340 (11.1)	1820 (8.9)	28 520 (11.3)
Administrative health region			
Erie St Clair	14 471 (5.3)	761 (3.7)	13 710 (5.4)
South West	19 222 (7.0)	1370 (6.7)	17 852 (7.1)
Waterloo Wellington	14 747 (5.4)	1304 (6.4)	13 443 (5.3)
Hamilton Niagara Haldimand Brant	29 484 (10.8)	1922 (9.4)	27 562 (10.9)
Central West	19 876 (7.3)	1055 (5.2)	18 821 (7.4)
Mississauga Halton	22 566 (8.3)	1722 (8.4)	20 844 (8.2)
Toronto Central	21 698 (7.9)	2308 (11.3)	19 390 (7.7)
Central	35 676 (13.0)	2610 (12.8)	33 066 (13.1)
Central East	32 100 (11.7)	2547 (12.4)	29 553 (11.7)
South East	12 044 (4.4)	908 (4.4)	11 136 (4.4)
Champlain	25 708 (9.4)	2223 (10.9)	23 485 (9.3)
North Simcoe Muskoka	8913 (3.3)	739 (3.6)	8174 (3.2)
North East	11 085 (4.1)	615 (3.0)	10 470 (4.1)
North West	5900 (2.2)	379 (1.9)	5521 (2.2)
**Clinical characteristics**			
Physical health condition			
Asthma	62 683 (22.9)	6195 (30.3)	56 488 (22.3)
Cancer	2850 (1.0)	49 (0.2)	2801 (1.1)
Cerebral palsy	6268 (2.3)	206 (1.0)	6062 (2.4)
Congenital heart disease	19 904 (7.3)	1169 (5.7)	18 735 (7.4)
Crohn disease or colitis	1455 (0.5)	19 (0.1)	1436 (0.6)
Diabetes	4575 (1.7)	140 (0.7)	4435 (1.8)
Epilepsy	16 859 (6.2)	1433 (7.0)	15 426 (6.1)
Spina bifida	1851 (0.7)	65 (0.3)	1786 (0.7)
Chronic physical conditions, No.			
0	179 452 (65.6)	12 510 (61.1)	166 942 (66.0)
1	75 641 (27.7)	6785 (33.2)	68 856 (27.2)
≥2	18 397 (6.7)	1168 (5.7)	17 229 (6.8)
Mental and/or behavioral health condition			
ADHD	22 249 (8.1)	7329 (35.8)	14 920 (5.9)
Mood and/or anxiety disorders	82 384 (30.1)	19 417 (94.9)	62 967 (24.9)
Autism spectrum disorder	12 029 (4.4)	2878 (14.1)	9151 (3.6)
Severe eating disorders	945 (0.3)	783 (3.8)	162 (0.1)
Schizophrenia spectrum disorders	1388 (0.5)	1121 (5.5)	267 (0.1)
Chronic mental and/or behavioral health conditions, No.			
0	185 261 (67.7)	434 (2.1)	184 827 (73.0)
1	61 444 (22.5)	10 435 (51.0)	51 009 (20.2)
≥2	26 785 (9.8)	9594 (46.9)	17 191 (6.8)
Chronic physical, mental, and/or behavioral conditions, No.			
0	132 363 (48.4)	282 (1.4)	132 081 (52.2)
1	75 998 (27.8)	6715 (32.8)	69 283 (27.4)
≥2	65 129 (23.8)	13 466 (65.8)	51 663 (20.4)

Abbreviation: ADHD, attention-deficit/hyperactivity disorder.

^a^ Data are based on administrative health care data housed at ICES, Toronto, Ontario, Canada, and are presented as the number (percentage) of patients unless otherwise indicated.

^b^ Costs are in 2021 Canadian dollars.

^c^ Quintile 1 was the lowest, and quintile 5 was the highest.

**Table 2.** Mean Health Care Costs for Children and Adolescents With High Health Care Costs in Ontario by Group and Health Service, 2012

Health care service	Cost, mean (95% CI), CAD^a^						
	All patients	Patients with high mental health care costs			Patients with high non–mental health care costs		
		Total	Mental health care	Non–mental health care	Total	Mental health care	Non–mental health care
Psychiatric hospitalizations	361.30 (348.30-374.30)	4729.20 (4567.90-4890.60)	4729.20 (4567.90-4890.60)	NA	8.10 (6.30-9.80)	8.10 (6.30-9.80)	NA
Acute medical hospitalizations	3159.30 (3093.20-3225.50)	143.50 (129.90-157.20)	NA	143.50 (129.90-157.20)	3403.20 (3331.80-3474.60)	NA	3403.20 (3331.80-3474.60)
Other hospital- or institution-based care^b^	20.00 (13.60-26.40)	0	NA	0	21.60 (14.70-28.60)	NA	21.60 (14.70-28.60)
Hospital outpatient clinic visits	916.60 (910.40-922.70)	1708.60 (1661.90-1755.40)	1530.50 (1485.90-1575.00)	178.20 (170.50-185.90)	852.50 (847.10-857.90)	33.90 (33.00-34.80)	818.60 (813.40-823.80)
Emergency department visits	328.80 (326.90-330.70)	437.50 (428.10-446.90)	225.60 (219.9-231.4)	211.90 (206.30-217.50)	320.00 318.10-321.90)	5.80 (5.60-6.00)	314.30 (312.40-316.10)
Other ambulatory care^c^	423.40 (417.50-429.20)	25.00 (22.00-28.10)	NA	25.00 (22.00-28.10)	455.60 (449.30-461.90)	NA	455.60 (449.30-461.90)
Physician services	1712.50 (1698.30-1726.60)	2740.60 (2680.50-2800.70)	2106.10 (2058.60-2153.60)	634.50 (604.30-664.80)	1629.30 (1614.90-1643.70)	63.80 (62.70-64.90)	1565.50 (1551.30-1579.80)
Outpatient prescription drugs^d^	226.70 (213.10-240.30)	188.60 (179.10-198.10)	110.20 (103.00-117.40)	78.40 (74.30-82.50)	229.80 (215.10-244.50)	10.50 (10.00-11.00)	219.30 (204.60-233.90)
Home care	787.90 (762.20-813.60)	67.10 (61.40-72.90)	0	67.10 (61.40-72.90)	846.20 (818.50-874.00)	0	846.20 (818.50-874.00)
Total	7936.40 (7850.30-8022.40)	10 040.20 (9822.80-10 257.50)	8701.50 (8504.30-8898.80)	1338.60 (1298.90-1378.40)	7766.20 (7674.90-7857.50)	122.00 (119.10-124.90)	7644.30 (7553.20-7735.10)

Abbreviation: NA, not applicable.

^a^ Costs are in 2021 Canadian dollars. Data are based on administrative health care data housed at ICES, Toronto, Ontario, Canada.

^b^ Includes inpatient rehabilitation, complex continuing care, and long-term care.

^c^ Includes same-day surgery, dialysis clinic visits, and cancer clinic visits.

^d^ Includes outpatient prescription drugs for individuals covered under the public provincial drug plan.

Patients with high mental health care costs (20 463 [7.5%]) had a mean cost of $10 040.20 (95% CI, $9822.80-$10 257.50), 23% higher than the cost for patients with high non–mental health costs ($7766.20; 95% CI, $7674.90-$7857.50). The subgroup with high mental health care costs had roughly the same number of females (48.6%) and males (51.4%) but was older than the general population with high health care costs (mean [SD] age, 12.84 [3.81] years). Most were long-term residents (94.8%) living in urban areas (90.8%) but were slightly more concentrated in high-income neighborhoods (21.6% in quintile 4 and 22.3% in quintile 5). This group had more chronic conditions, of which mood and/or anxiety disorders (94.9%), ADHD (35.8%), and asthma (30.3%) were the most prevalent. Costs were mostly for psychiatric hospitalizations (47.1%), physician care (27.3%), and mental health–related care (86.7%). For patients with high non–mental health care costs, mood and/or anxiety disorders (24.9%) and asthma (22.3%) were the most common chronic conditions; costs were mainly due to acute medical hospitalizations (43.8%), physician care (21.0%), and care not related to mental health (98.4%).

Few children and adolescents with high mental health care costs persisted in the high-cost state for more than 3 years (19%) (eTable 2 in Supplement 1); 710 patients in the total sample (3.5%) were lost to attrition. Patients in the moderate and persistent groups were younger and were more concentrated in high-income neighborhoods (Table 3). The prevalence of physical chronic conditions was roughly the same across all 3 subgroups. However, increased prevalence of ADHD and autism was associated with increased persistence; the opposite was found for schizophrenia spectrum disorders.

**Table 3.** Characteristics of Children and Adolescents With High Mental Health Care Costs in Ontario by High-Cost Status, 2012

Characteristic	Patients^a^			
	All (N = 20 463)	Sporadic (n = 16 585)	Moderate (n = 3416)	Persistent (n = 462)
Sex				
Female	9953 (48.6)	8172 (49.3)	1620 (47.4)	161 (34.8)
Male	10 510 (51.4)	8413 (50.7)	1796 (52.6)	301 (65.2)
Age, mean (SD), y	12.84 (3.81)	13.22 (3.88)	11.52 (2.97)	8.85 (2.24)
Migrant status				
Long-term resident	19 407 (94.8)	15 688 (94.6)	3267 (95.6)	452 (97.8)
Immigrant	860 (4.2)	729 (4.4)	123 (3.6)	8 (1.7)
Refugee	196 (1.0)	≤170 (1.0)	≤30 (0.8)	≤5 (0.4)
Neighborhood income quintile^b^				
Missing	138 (0.7)	≤120 (0.7)	≤20 (0.5)	≤5 (0.2)
1	3856 (18.8)	3141 (18.9)	622 (18.2)	93 (20.1)
2	3586 (17.5)	2913 (17.6)	585 (17.1)	88 (19.0)
3	3907 (19.1)	3222 (19.4)	624 (18.3)	61 (13.2)
4	4416 (21.6)	3602 (21.7)	711 (20.8)	103 (22.3)
5	4560 (22.3)	3588 (21.6)	856 (25.1)	116 (25.1)
Rural residence				
Missing	61 (0.3)	≤50 (0.3)	≤15 (0.3)	≤5 (0.2)
Yes	1820 (8.9)	1527 (9.2)	274 (8.0)	19 (4.1)
No	18 582 (90.8)	15 009 (90.5)	3131 (91.7)	442 (95.7)
Administrative health region				
Erie St Clair	761 (3.7)	643 (3.9)	106 (3.1)	12 (2.6)
South West	1370 (6.7)	1108 (6.7)	235 (6.9)	27 (5.8)
Waterloo Wellington	1304 (6.4)	1087 (6.6)	192 (5.6)	25 (5.4)
Hamilton Niagara Haldimand Brant	1922 (9.4)	1592 (9.6)	286 (8.4)	44 (9.5)
Central West	1055 (5.2)	903 (5.4)	140 (4.1)	12 (2.6)
Mississauga Halton	1722 (8.4)	1375 (8.3)	291 (8.5)	56 (12.1)
Toronto Central	2308 (11.3)	1733 (10.4)	512 (15.0)	63 (13.6)
Central	2610 (12.8)	2135 (12.9)	432 (12.6)	43 (9.3)
Central East	2547 (12.4)	2062 (12.4)	429 (12.6)	56 (12.1)
South East	908 (4.4)	733 (4.4)	156 (4.6)	19 (4.1)
Champlain	2223 (10.9)	1707 (10.3)	431 (12.6)	85 (18.4)
North Simcoe Muskoka	739 (3.6)	638 (3.8)	93 (2.7)	8 (1.7)
North East	615 (3.0)	531 (3.2)	77 (2.3)	7 (1.5)
North West	379 (1.9)	≤340 (2.0)	≤40 (1.1)	≤5 (1.1)
Chronic condition				
Asthma	6195 (30.3)	5002 (30.2)	1052 (30.8)	141 (30.5)
Cancer	49 (0.2)	≤45 (0.3)	≤5 (0.1)	≤5 (0.2)
Cerebral palsy	206 (1.0)	162 (1.0)	37 (1.1)	7 (1.5)
Congenital heart disease	1169 (5.7)	923 (5.6)	217 (6.4)	29 (6.3)
Crohn disease or colitis	19 (0.1)	≤20 (0.1)	≤5 (0.1)	0
Diabetes	140 (0.7)	120 (0.7)	20 (0.6)	0
Epilepsy	1433 (7.0)	1141 (6.9)	262 (7.7)	30 (6.5)
Spina bifida	65 (0.3)	59 (0.4)	6 (0.2)	0
Chronic physical health conditions, No.				
0	12 510 (61.1)	10 170 (61.3)	2062 (60.4)	278 (60.2)
1	6785 (33.2)	5495 (33.1)	1127 (33.0)	163 (35.3)
≥2	1168 (5.7)	920 (5.5)	227 (6.6)	21 (4.5)
ADHD	7329 (35.8)	5571 (33.6)	1503 (44.0)	255 (55.2)
Mood and/or anxiety disorders	19 417 (94.9)	15 687 (94.6)	3282 (96.1)	448 (97.0)
Autism spectrum disorder	2878 (14.1)	2090 (12.6)	653 (19.1)	135 (29.2)
Severe eating disorders	783 (3.8)	611 (3.7)	163 (4.8)	9 (1.9)
Schizophrenia spectrum disorders	1121 (5.5)	942 (5.7)	172 (5.0)	7 (1.5)
Chronic mental and/or behavioral health conditions, No.				
0	434 (2.1)	385 (2.3)	43 (1.3)	6 (1.3)
1	10 435 (51.0)	8869 (53.5)	1420 (41.6)	146 (31.6)
≥2	9594 (46.9)	7331 (44.2)	1953 (57.2)	310 (67.1)
Chronic physical, mental, and/or behavioral health conditions, No.				
0	282 (1.4)	≤260 (1.6)	≤25 (0.6)	≤5 (0.2)
1	6715 (32.8)	5721 (34.5)	901 (26.4)	93 (20.1)
≥2	13 466 (65.8)	10 604 (63.9)	2494 (73.0)	368 (79.7)

Abbreviation: ADHD, attention-deficit/hyperactivity disorder.

^a^ Data are based on administrative health care data housed at ICES, Toronto, Ontario, Canada, and are presented as the number (percentage) of patients unless otherwise indicated. Standardized differences greater than 0.1 are considered clinically relevant.

^b^ Quintile 1 was the lowest, and quintile 5 was the highest.

Costs were distributed differently across subgroups. Costs for patients with sporadic and moderate high costs were mostly due to psychiatric hospitalizations (48.2% and 44.6%, respectively) and physician care (26.8% and 28.4%, respectively); this also was found for patients with persistent high costs but with a different breakdown (35.0% for physician care and 29.0% for psychiatric hospitalizations). Among patients with sporadic high costs, costs were high in the first year only and then decreased over time (Figure and eTable 3 in Supplement 1). Patients with moderate high costs had higher costs in the first 3 years (and higher than among patients with persistent high costs), decreasing thereafter, while patients with persistent high costs had high costs across all 8 years (though lower than patients with moderate high costs for the first 3 years).

**Figure.**
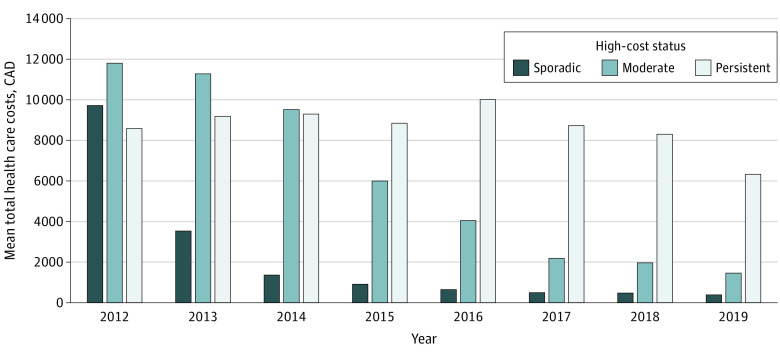
Mean Total Health Care Costs for 20 463 Children and Adolescents With High Mental Health Care Costs in Ontario, Canada, by High-Cost Status, 2012-2019 Based on administrative health care data housed at ICES, Toronto, Ontario, Canada. Costs are in 2021 Canadian dollars.

The risk of persistent high-cost status compared with sporadic high-cost status was higher for younger patients except those aged 0 to 3 years (ages 4-8 years: RRR, 3.29; 95% CI, 1.57-6.90; ages 9-13 years: RRR, 3.47; 95% CI, 1.58-7.65) but was lower for males (RRR, 0.79; 95% CI, 0.64-0.98) and rural dwellers (RRR, 0.58; 95% CI, 0.39-0.86) (Table 4). The highest RRRs observed were for mood and/or anxiety disorders (6.17; 95% CI, 3.19-11.96) and schizophrenia spectrum disorders (2.98; 95% CI, 2.14-4.14) (Table 4). Similar associations with the risk of having moderate rather than sporadic high-cost status were found for these variables. Findings were largely unchanged with the exclusion of attritors (eTable 4 in Supplement 1).

**Table 4.** Pooled Multinomial Logistic Regression of Factors Associated With Persistency in the High-Cost State Among Children and Adolescents With High Mental Health Care Costs in Ontario, 2012-2019

Factor	Ratio of relative risk (95% CI)^a^	
	Persistent vs sporadic high costs	Moderate vs sporadic high costs
Sex		
Female	1 [Reference]	1 [Reference]
Male	0.79 (0.64-0.98)	0.76 (0.70-0.83)
Age group, y		
0-3	1 [Reference]	1 [Reference]
4-8	3.29 (1.57-6.90)	2.48 (1.74-3.52)
9-13	3.47 (1.58-7.65)	3.61 (2.45-5.31)
14-17	1.02 (0.46-2.27)	2.57 (1.74-3.79)
≥18	0.06 (0.02-0.13)	0.81 (0.54-1.20)
Migrant status		
Long-term resident	1 [Reference]	1 [Reference]
Immigrant	0.51 (0.25-1.03)	0.88 (0.72-1.08)
Refugee	0.61 (0.14-2.57)	0.89 (0.59-1.35)
Neighborhood income quintile^b^		
1	1 [Reference]	1 [Reference]
2	1.02 (0.81-1.28)	1.03 (0.95-1.13)
3	1.09 (0.86-1.39)	1.07 (0.97-1.17)
4	1.04 (0.81-1.33)	1.08 (0.98-1.19)
5	1.08 (0.84-1.38)	1.16 (1.06-1.28)
Missing	0.43 (0.06-3.11)	0.70 (0.36-1.36)
Rural residence		
Yes	0.58 (0.39-0.86)	0.92 (0.80-1.04)
No	1 [Reference]	1 [Reference]
Missing	2.51 (0.31-20.09)	1.62 (0.80-3.29)
Asthma	1.02 (0.83-1.25)	1.05 (0.97-1.14)
Cancer	0.94 (0.16-5.41)	0.72 (0.37-1.41)
Chronic physical, mental, and/or behavioral health conditions^c^		
Cerebral palsy	1.15 (0.52-2.55)	1.02 (0.71-1.45)
Congenital heart disease	1.18 (0.82-1.71)	1.13 (0.97-1.31)
Crohn disease or colitis	0.33 (0.04-2.66)	0.64 (0.26-1.57)
Diabetes	0.58 (0.25-1.34)	1.22 (0.92-1.61)
Epilepsy	1.09 (0.78-1.52)	1.21 (1.06-1.37)
Spina bifida	0.37 (0.05-2.81)	0.57 (0.28-1.16)
ADHD	2.46 (2.03-2.99)	1.66 (1.54-1.80)
Mood and/or anxiety disorders	6.17 (3.19-11.96)	2.41 (1.90-3.06)
Autism spectrum disorder	2.31 (1.89-2.82)	1.63 (1.48-1.80)
Severe eating disorders	2.13 (1.23-3.67)	2.39 (2.05-2.80)
Schizophrenia spectrum disorders	2.98 (2.14-4.14)	1.83 (1.61-2.07)
Administrative health region		
Erie St Clair	0.63 (0.36-1.12)	0.51 (0.41-0.64)
South West	0.68 (0.42-1.09)	0.73 (0.61-0.87)
Waterloo Wellington	0.60 (0.38-0.95)	0.53 (0.45-0.64)
Hamilton Niagara Haldimand Brant	0.82 (0.57-1.19)	0.57 (0.49-0.67)
Central West	0.29 (0.16-0.53)	0.50 (0.41-0.61)
Mississauga Halton	1.16 (0.80-1.68)	0.74 (0.63-0.86)
Toronto Central	1 [Reference]	1 [Reference]
Central	0.45 (0.30-0.67)	0.61 (0.53-0.71)
Central East	0.81 (0.57-1.17)	0.75 (0.65-0.86)
South East	0.68 (0.41-1.13)	0.68 (0.56-0.83)
Champlain	1.16 (0.82-1.63)	0.78 (0.67-0.90)
North Simcoe Muskoka	0.37 (0.20-0.69)	0.50 (0.40-0.62)
North East	0.55 (0.25-1.21)	0.55 (0.43-0.70)
North West	0.56 (0.22-1.45)	0.40 (0.29-0.57)
Year		
2012	1 [Reference]	1 [Reference]
2013	1.16 (1.14-1.18)	1.09 (1.08-1.10)
2014	1.40 (1.36-1.45)	1.24 (1.22-1.26)
2015	1.71 (1.64-1.78)	1.41 (1.37-1.44)
2016	2.18 (2.07-2.30)	1.61 (1.56-1.66)
2017	2.78 (2.61-2.97)	1.79 (1.72-1.86)
2018	3.55 (3.29-3.85)	1.95 (1.86-2.04)
2019	4.56 (4.15-5.01)	2.09 (1.99-2.19)

Abbreviation: ADHD, attention-deficit/hyperactivity disorder.

^a^ Data are based on administrative health care data housed at ICES, Toronto, Ontario, Canada.

^b^ Quintile 1 was the lowest, and quintile 5 was the highest.

^c^ The reference group for each condition was absence of the condition.

## Discussion

In this study, most children and adolescents with high health care costs were young (aged 0-3 years) and resided in urban neighborhoods. The most common chronic conditions were asthma and mental disorders, and most costs were due to acute medical hospitalizations and physician care. Patients with high mental health care costs had characteristics mostly similar to those of patients with high health care costs but were older in general. The most common chronic conditions were mood and/or anxiety disorders, ADHD, and asthma. Costs for this group were 23% higher than for patients with non–mental health care costs and were mostly due to psychiatric hospitalizations and physician care. Importantly, few individuals persisted in the high-cost state; most who did had mental and/or behavioral disorders. In the regression analysis, mood and/or anxiety and schizophrenia spectrum disorders were identified as the main factors associated with persistence in the high-cost state.

Few studies have examined children and adolescents with high health care costs, and even fewer have specifically examined those with mental disorders, which is likely why the latter have been termed as “unseen high-cost users.”^5^ This is not surprising as some of the mental health care that children and adolescents receive falls outside the health care system. Our results are in line with prior work by some of us on adults with high health care costs.^9,10,14^ That work also revealed that adult patients with high mental health care costs had 30% higher mean costs than adult patients with high non–mental health care costs and a higher proportion of mental disorders than the latter. However, adults tended to persist in the high-cost state, and adults with persistent high costs had higher costs for all years compared with the other 2 patient subgroups. A novel and positive finding of the current study was that few children and adolescents remained in the high-cost state for many years. Nonetheless, they should be monitored over time to ensure they receive appropriate care to prevent them from becoming patients with high costs in adulthood.

Our findings are also in line with other work examining children and adolescents with high health care costs^1,6,7^ that found that mental and behavioral disorders were common among the general youth population with high costs. Wodchis and colleagues^1^ found that the most frequent diagnoses among the top 1% of the cost distribution in Ontario, Canada, included preterm birth, cancer, and mental disorders. Using data on privately insured individuals aged 13 to 21 years in Massachusetts who incurred high costs, Gray and colleagues^6^ found that mental disorders were the most common diagnosis, 59% of patients with high costs had at least 1 behavioral health diagnosis, and characteristics associated with having high health care costs included having 1 or more complex chronic conditions, any behavioral health diagnosis, and obesity. Using 2016 Medicaid claims on patients aged 3 to 17 years with mental health conditions, Doupnik and colleagues^7^ found that children with mental health conditions accounted for 55% of spending and that patients in the highest spending group were older and had multiple mental health conditions and complex chronic physical health conditions, with most of the costs due to general hospitalizations.

Access to outpatient mental health care has typically been poor for children and adolescents^30,31^ and has likely worsened with the COVID-19 pandemic. To ensure that children and adolescents with mental and behavioral disorders receive timely care, it is important to address wait times.^32,33^ Moreover, better integration and coordination of clinical and social care may help address children’s and adolescents’ mental health needs, which could be achieved without significant additional investments by adopting similar health care delivery models used in other pediatric care settings (eg, oncology).^5^ The implementation of integrated youth services, an emerging model of care offering a broad range of mental health and social services for children and adolescents in 1 location, could help in this regard.^34^ This networked “one-stop-shop” service delivery model provides children and adolescents with rapid access to high-quality, evidence-based, integrated mental health and substance use services with a focus on early intervention.^34^ Prior research suggests that integrating mental health care professionals into pediatric primary care could prove to be cost-effective if hospitalizations can be reduced.^6^ Finally, incorporating mental health services into routine care for children with chronic physical illnesses may be associated with improved quality of life and cost savings due to fewer disease exacerbations requiring hospitalization.^7^

We used a population-based sample of all children and adolescents with high health care costs in a large Canadian province; previous work has examined specific subpopulations (privately insured,^6^ Medicaid insured^4,7^) or used small samples. We examined all children and adolescents with high costs, with a particular focus on those who used mostly mental health care. Furthermore, we observed a cohort of patients with high costs who had mental illness over many years.

### Limitations

This study has some limitations. We examined costs from a health system perspective, which captures only some of the care provided to children and adolescents. We were unable to account for costs of mental health care provided in schools and community-based agencies and care provided by social workers and psychologists. Thus, our cost estimates are likely an underestimate of the total cost incurred by children and adolescents with high costs. We were able to examine only chronic conditions for which there were validated algorithms or disease-specific physician billing codes in the data. For example, we were not able to examine conduct disorder. Furthermore, due to the lack of ethnicity and race information in the Ontario administrative data, we were not able to characterize patients by ethnicity or race, as done elsewhere.^11^

## Conclusions

In this cohort study of children and adolescents with high health care costs, some patients with high costs had high levels of mental health care use. Moreover, patients with high levels of mental health care use had higher mean costs of care than those who did not have high levels of mental health care use. However, an important finding is that few children and adolescents persisted in the high-cost state for many years, contrary to what has been found for adults with high costs.^14^ These findings may be important for informing the development of care coordination interventions and service delivery models of care, such as youth integrated services, to reduce costs and improve outcomes for this patient population.

## Notes

Supplement 1. **eTable 1.** Summary of Administrative Health Care Databases Employed in the Analysis **eTable 2.** Years in the High-Cost State Among Children and Adolescents With High Mental Health Costs in Ontario by Nonattrition and Attrition Cohorts, 2012-2019 **eTable 3.** Mean Health Care Costs and 95% CIs by Group of Patients With High Costs and by Health Service, 2012-2019 **eTable 4.** Pooled Multinomial Logistic Regression of Factors Associated With Persistency in the High-Cost State Among Patients With High Mental Health Costs in Ontario, 2012-2019, Excluding Decedents and Individuals Ineligible for Public Health Care Insurance Coverage

Supplement 2.
Data Sharing Statement
