# Supplementary material for: Mental Health Care Use Among Children and Adolescents With High Health Care Costs in Ontario, Canada
Source: JAMA Netw Open. 2023 May 12;6(5):e2313172. doi: 10.1001/jamanetworkopen.2023.13172 (PMC10182426; doi:10.1001/jamanetworkopen.2023.13172)
Supplement: Supplement 1. — eTable 1. Summary of Administrative Health Care Databases Employed in the Analysis eTable 2. Years in the High-Cost State Among Children and Adolescents With High Mental Health Costs in Ontario by Nonattrition and Attrition Cohorts, 2012-2019 eTable 3. Mean Health Care Costs and 95% CIs by Group of Patients With High Costs and by Health Service, 2012-2019 eTable 4. Pooled Multinomial Logistic Regression of Factors Associated With Persistency in the High-Cost State Among Patients With High Mental Health Costs in Ontario, 2012-2019, Excluding Decedents and Individuals Ineligible for Public Health Care Insurance Coverage [file jamanetwopen-e2313172-s001.pdf]

Supplementary Online Content

de Oliveira C, Iwajomo T, Kurdyak P. Mental health care use among children and adolescents with high health care costs in Ontario, Canada. *JAMA Netw Open*. 2023;6(5):e2313172. doi:10.1001/jamanetworkopen.2023.13172

eTable 1. Summary of Administrative Health Care Databases Employed in the Analysis
eTable 2. Years in the High-Cost State Among Children and Adolescents With High Mental Health Costs in Ontario by Nonattrition and Attrition Cohorts, 2012-2019
eTable 3. Mean Health Care Costs and 95% CIs by Group of Patients With High Costs and by Health Service, 2012-2019
eTable 4. Pooled Multinomial Logistic Regression of Factors Associated With Persistency in the High-Cost State Among Patients With High Mental Health Costs in Ontario, 2012-2019, Excluding Decedents and Individuals Ineligible for Public Health Care Insurance Coverage

This supplementary material has been provided by the authors to give readers additional information about their work.

© 2023 de Oliveira C et al. *JAMA Network Open*.

eTable 1. Summary of administrative health care databases employed in the analysis

Database	Setting	Description
Discharge Abstract Database (DAD)	acute medical inpatient hospitalisations	The DAD is a national database that contains demographic and clinical data on inpatient hospital admissions.
Ontario Mental Health Reporting System (OMHRS)	psychiatric inpatient hospitalisations	The OMHRS contains demographic and clinical data on all psychiatric hospitalisations in designated adult psychiatric beds in Ontario. Psychiatric hospitalisations in non-psychiatric designated beds are captured in the DAD.
Continuing Care Reporting System (CCRS)	complex continuing care, long-term care	The CCRS contains demographic and clinical data on individuals receiving facility-based continuing care. These services include medical long-term care, rehabilitation, geriatric assessment, respite palliative care, and nursing home care.
National Rehabilitation Reporting System (NRS)	rehabilitation	The NRS contains national data on rehabilitation facilities and clients, collected from participating adult inpatient rehabilitation facilities and programs.
National Ambulatory Care Reporting System (NACRS)	emergency department visits, day surgery and outpatient clinics	The NACRS contains data on all ambulatory care including emergency department visits, day surgery and outpatient clinic visits (such as chemotherapy and dialysis clinic visits).
Ontario Health Insurance Plan (OHIP) Claims Database	outpatient and physician services	The OHIP claims database covers all services and procedures provided by health care providers who can claim under OHIP (e.g., physician and laboratory/diagnostic services).
Ontario Drug Benefit (ODB) Claims Database	outpatient prescription drugs	The ODB claims database includes data on all drugs dispensed in community pharmacies and long-term care/nursing facilities. The ODB program covers prescription drugs listed in the provincial formulary for all seniors (aged 65+) and those under the age of 65 years living in a long-term care home, a home for special care or a Community Home for Opportunity, receiving professional home and community care services, enrolled in the Trillium Drug Program, or on social assistance.
Home Care Database (HCD)	home care	The HCD provides data on government-funded services coordinated by Ontario's Community Care Access Centres for individuals requiring home care.
Registered Persons Database (RPDB)	---	The RPDB is population-based registry, which contains demographic information such as sex and age, on all persons registered under the Ontario Health Insurance Plan.
Immigration, Refugees, and Citizenship Canada (IRCC) Database	---	The IRCC database includes information on permanent and temporary residents as well as immigration and citizenship programs.

© 2023 de Oliveira C et al. *JAMA Network Open*.

Census data	---	The Census data provides information about people and housing units in Canada by their demographic, social and economic characteristics.

© 2023 de Oliveira C et al. *JAMA Network Open*.

eTable 2. Number of years in the high-cost state among mental health high-cost children and adolescents (n = 20,463) in Ontario broken down by non-attrition and attrition cohorts, 2012-2019

	All			Non-attrition cohort			Attrition cohort		
**Years in high-cost state**	**N**	**Col%**	**Row%**	**N**	**Col%**	**Row%**	**N**	**Col%**	**Row%**
**1**	11,989	58.59	100	11,493	58.18	95.86	496	69.86	4.14
**2**	4,596	22.46	100	4,452	22.54	96.87	144	20.28	3.13
**3**	1,977	9.66	100	1,940	9.82	98.13	37	5.21	1.87
**4**	965	4.72	100	946	4.79	98.03	19	2.68	1.97
**5**	474	2.32	100	<=465	2.35	97.89	<=10	1.41	2.11
**6**	264	1.29	100	<=265	1.32	98.48	<=5	0.56	1.52
**7**	140	0.68	100	140	0.71	100	0	.	.
**8**	58	0.28	100	58	0.29	100	0	.	.
**Total**	20,463	100	100	19,753	100	96.53	710	100	3.47

Note: The attrition cohort refers to the cohort of patients who did not have data for the entire analysis period (i.e., for all 8 years) either due to loss to follow up (individuals who died, moved out of the province or those who were no longer eligible for public health care insurance in Ontario). The non-attrition cohort refers to the cohort of patients who had data for every year of the analysis (i.e., for all 8 years).

Source: Administrative health care data housed at ICES, Toronto Ontario

© 2023 de Oliveira C et al. *JAMA Network Open*.

eTable 3. Mean health care costs (and 95% confidence intervals) by high-cost patient group and health service, 2012-2019

	**Sporadic** n=16,585	**Lower CI**	**Upper CI**	**Moderate** n=3,416	**Lower CI**	**Upper CI**	**Persistent** n=462	**Lower CI**	**Upper CI**
**Psychiatric hospitalisations**									
**2012**	4,681.70	4,504.30	4,859.00	5,263.10	4,833.40	5,692.70	2,489.40	1,853.20	3,125.50
**2013**	978.10	883.60	1,072.70	4,483.40	3,990.80	4,976.00	2,478.10	1,749.30	3,206.90
**2014**	132.40	109.20	155.70	4,035.10	3,526.90	4,543.30	2,819.20	2,020.10	3,618.30
**2015**	68.60	51.70	85.60	2,337.90	1,967.30	2,708.50	2,854.50	1,891.30	3,817.70
**2016**	38.70	25.20	52.20	1,424.20	1,099.20	1,749.20	3,606.20	2,208.90	5,003.60
**2017**	40.90	15.60	66.20	579.60	455.10	704.10	2,948.40	2,075.90	3,820.80
**2018**	18.30	8.40	28.30	494.60	343.80	645.30	2,856.60	1,742.50	3,970.70
**2019**	37.70	14.30	61.10	500.10	233.30	767.00	2,681.80	1,186.00	4,177.60
**Acute care hospitalisations**									
**2012**	146.60	131.40	161.80	134.00	101.20	166.90	102.70	3.20	202.20
**2013**	208.40	171.50	245.30	301.40	222.60	380.10	81.30	28.50	134.10
**2014**	138.80	112.60	165.10	244.50	177.90	311.20	155.30	59.10	251.50
**2015**	111.80	84.70	138.90	229.70	167.00	292.30	116.20	35.50	196.90
**2016**	68.30	48.00	88.50	199.90	124.80	275.00	235.60	91.40	379.70
**2017**	44.30	29.80	58.80	160.70	80.10	241.30	220.70	106.30	335.20
**2018**	42.30	24.00	60.70	103.70	49.10	158.30	113.40	39.90	187.00
**2019**	66.40	32.00	100.80	111.60	39.80	183.50	158.30	59.80	256.80
**Other hospital/institution-based care***									
**2012**	0.00	.	.	0.00	.	.	0.00	.	.
**2013**	0.70	-0.70	2.20	0.00	.	.	0.00	.	.
**2014**	0.40	-0.30	1.00	0.00	.	.	0.00	.	.
**2015**	0.00	.	.	0.60	-0.60	1.80	0.00	.	.
**2016**	0.00	.	.	8.90	-8.50	26.30	0.00	.	.
**2017**	0.00	.	.	0.00	.	.	0.00	.	.

© 2023 de Oliveira C et al. *JAMA Network Open*.

**2018**	0.00	.	.	0.00	.	.	0.00	.	.
**2019**	3.50	-1.50	8.60	0.00	.	.	0.00	.	.
**Hospital outpatient clinic visits**									
**2012**	1,597.60	1,550.60	1,644.70	2,213.90	2,055.70	2,372.00	1,957.80	1,725.30	2,190.30
**2013**	634.20	607.60	660.70	2,280.10	2,111.60	2,448.50	2,004.20	1,714.00	2,294.40
**2014**	198.80	189.10	208.50	1,371.30	1,277.60	1,464.90	1,599.20	1,375.50	1,823.00
**2015**	118.00	110.70	125.30	748.90	701.70	796.00	1,398.90	1,210.10	1,587.70
**2016**	85.70	79.70	91.60	495.20	451.90	538.40	1,465.90	1,239.40	1,692.40
**2017**	59.90	55.00	64.70	273.90	247.60	300.10	1,133.70	994.10	1,273.20
**2018**	50.40	44.90	55.90	184.10	162.40	205.70	876.00	754.60	997.30
**2019**	41.70	37.40	46.10	158.00	135.40	180.70	720.60	592.90	848.20
**Emergency department visits**									
**2012**	450.00	439.50	460.50	402.90	379.80	426.10	245.10	203.70	286.50
**2013**	210.20	200.90	219.50	406.30	377.40	435.20	292.60	230.60	354.50
**2014**	111.70	106.60	116.70	432.50	399.20	465.80	333.70	269.80	397.70
**2015**	73.00	68.60	77.40	312.90	282.10	343.70	309.40	249.20	369.50
**2016**	51.30	48.00	54.70	249.30	214.60	284.10	422.40	339.20	505.50
**2017**	35.70	32.90	38.50	150.30	129.30	171.40	471.10	380.60	561.50
**2018**	27.50	25.10	29.90	105.20	91.50	118.80	407.80	290.70	525.00
**2019**	24.40	21.90	26.80	78.70	66.90	90.60	345.80	243.00	448.60
**Other ambulatory care****									
**2012**	26.40	22.90	29.90	19.30	13.10	25.60	18.00	1.60	34.50
**2013**	49.50	40.90	58.00	44.00	33.80	54.10	32.60	6.80	58.50
**2014**	38.90	33.50	44.20	41.10	30.90	51.20	14.50	2.80	26.10
**2015**	26.50	20.00	33.10	42.90	33.50	52.30	30.80	10.50	51.10
**2016**	27.20	18.60	35.80	38.40	28.90	48.00	27.50	5.30	49.80
**2017**	27.10	12.80	41.40	24.50	17.50	31.50	35.80	9.10	62.60
**2018**	21.30	5.50	37.20	19.80	13.20	26.40	52.00	21.80	82.10
**2019**	14.30	6.80	21.80	18.60	11.60	25.50	33.80	12.00	55.60

© 2023 de Oliveira C et al. *JAMA Network Open*.

**Physician services**									
**2012**	2,606.70	2,542.50	2,670.80	3,354.80	3,179.10	3,530.50	3,008.10	2,744.20	3,272.10
**2013**	1,252.90	1,179.70	1,326.20	3,250.90	3,086.70	3,415.20	3,262.50	2,941.10	3,583.80
**2014**	588.30	554.50	622.20	2,877.30	2,696.40	3,058.20	3,254.40	2,931.30	3,577.50
**2015**	382.60	369.10	396.00	1,891.90	1,799.30	1,984.50	3,085.20	2,787.40	3,382.90
**2016**	267.60	256.70	278.50	1,277.00	1,191.30	1,362.60	3,245.60	2,906.80	3,584.50
**2017**	195.80	186.10	205.40	774.60	720.20	828.90	2,941.10	2,659.70	3,222.40
**2018**	154.10	145.20	163.00	570.50	521.60	619.30	2,271.80	2,003.00	2,540.60
**2019**	130.50	121.50	139.60	403.30	366.50	440.10	1,652.30	1,434.60	1,870.00
**Outpatient prescription drugs*** **									
**2012**	152.00	142.70	161.30	314.10	283.70	344.60	575.10	463.40	686.70
**2013**	125.00	116.00	133.90	404.60	368.30	440.80	838.10	654.10	1,022.10
**2014**	87.90	79.40	96.50	431.90	393.20	470.50	956.00	748.40	1,163.70
**2015**	72.30	63.10	81.50	357.90	320.30	395.60	946.10	754.30	1,137.80
**2016**	55.30	46.70	63.80	281.90	247.40	316.40	918.40	754.70	1,082.20
**2017**	41.70	34.00	49.50	167.60	142.70	192.60	893.10	743.50	1,042.70
**2018**	124.90	113.20	136.60	445.70	398.10	493.20	1,661.00	1,480.70	1,841.30
**2019**	57.20	49.00	65.40	175.70	154.20	197.10	711.70	602.40	821.00
**Home care**									
**2012**	57.70	51.20	64.10	96.90	83.30	110.40	187.00	142.70	231.30
**2013**	74.60	66.90	82.30	106.00	91.70	120.40	190.90	144.10	237.80
**2014**	65.90	56.90	74.80	80.10	64.70	95.50	162.40	118.40	206.30
**2015**	57.30	47.10	67.60	71.70	53.20	90.10	102.60	68.40	136.90
**2016**	52.40	41.20	63.60	70.20	45.90	94.50	89.50	58.20	120.70
**2017**	48.80	29.10	68.50	54.40	29.00	79.70	82.20	46.90	117.60
**2018**	38.00	18.70	57.40	40.40	19.90	60.90	65.00	35.60	94.50
**2019**	13.40	7.00	19.90	13.10	-0.30	26.40	22.30	-9.10	53.80
**Total cost**									
**2012**	9,718.50	9,482.70	9,954.30	11,799.00	11,194.50	12,403.50	8,583.10	7,669.30	9,496.90

© 2023 de Oliveira C et al. *JAMA Network Open*.

**2013**	3,533.60	3,369.70	3,697.50	11,276.60	10,599.50	11,953.70	9,180.30	8,140.00	10,220.50
**2014**	1,363.10	1,292.30	1,433.90	9,513.60	8,826.70	10,200.50	9,294.60	8,162.50	10,426.70
**2015**	910.20	855.20	965.20	5,994.50	5,511.90	6,477.10	8,843.90	7,589.30	10,098.50
**2016**	646.40	601.90	691.00	4,045.10	3,601.60	4,488.60	10,011.30	8,305.60	11,716.90
**2017**	494.10	441.40	546.90	2,185.60	1,962.60	2,408.60	8,726.10	7,509.10	9,943.10
**2018**	476.80	427.60	526.00	1,963.80	1,737.50	2,190.10	8,303.40	6,875.00	9,731.80
**2019**	389.20	327.30	451.10	1,459.10	1,151.70	1,766.50	6,326.60	4,662.90	7,990.20

* includes rehabilitation, complex continuing care and long-term care
** includes same-day surgery, dialysis clinic visits and cancer clinic visits
*** includes outpatient prescriptions drugs for individuals covered under the public provincial drug plan

Note: Costs are in 2021 CAD.

Legend: CI – confidence interval

Source: Administrative health care data housed at ICES, Toronto Ontario

© 2023 de Oliveira C et al. *JAMA Network Open*.

eTable 4. Pooled multinomial logistic regression: predictors of persistency in the high-cost state among mental health high-cost patients (n = 19,753) in Ontario, 2012-2019 (excluding decedents and individuals ineligible for public health care insurance coverage)

**Predictor**	**Ratio of relative risk of persistent vs. sporadic high-cost patient**	**Lower CI**	**Upper CI**	**Ratio of relative risk of moderate vs. sporadic high-cost patient**	**Lower CI**	**Upper CI**
**Sex**						
Male	0.78	0.63	0.97	0.77	0.71	0.84
**Age group**						
4-8	4.22	1.90	9.34	2.46	1.73	3.50
9-13	4.40	1.90	10.22	3.57	2.42	5.26
14-17	1.28	0.55	3.01	2.55	1.72	3.77
18+	0.07	0.03	0.17	0.81	0.54	1.21
**Migrant status**						
Immigrant	0.51	0.25	1.03	0.89	0.73	1.09
Refugee	0.62	0.15	2.62	0.85	0.56	1.30
**Neighbourhood income quintile**						
2 – medium low	1.02	0.82	1.28	1.03	0.94	1.13
3 – medium	1.10	0.86	1.39	1.07	0.97	1.17
4 – medium high	1.02	0.80	1.31	1.08	0.98	1.19
5 – high	1.08	0.84	1.38	1.17	1.06	1.29
Missing	0.00*	0.00	0.00	0.72	0.37	1.40
**Rural residence**						
Yes	0.58	0.39	0.87	0.92	0.81	1.05
Missing	---	---	---	1.69	0.83	3.44
**Asthma**						
Yes	1.01	0.82	1.25	1.05	0.97	1.14
**Cancer**						
Yes	1.07	0.19	6.13	0.77	0.38	1.56

© 2023 de Oliveira C et al. *JAMA Network Open*.

**Cerebral palsy**						
Yes	1.14	0.52	2.53	1.00	0.70	1.43
**Congenital heart disease**						
Yes	1.18	0.82	1.70	1.12	0.97	1.30
**Crohn's/colitis**						
Yes	0.35	0.04	2.87	0.68	0.28	1.68
**Diabetes**						
Yes	0.58	0.25	1.34	1.20	0.91	1.60
**Epilepsy**						
Yes	1.08	0.77	1.51	1.22	1.07	1.38
**Spina bifida**						
Yes	0.37	0.05	2.80	0.57	0.28	1.16
**ADHD**						
Yes	2.49	2.05	3.03	1.66	1.53	1.79
**Mood and/or anxiety disorders**						
Yes	6.02	3.11	11.68	2.37	1.87	3.02
**Autism spectrum disorder**						
Yes	2.28	1.87	2.80	1.63	1.48	1.79
**Eating disorders (severe)**						
Yes	2.18	1.26	3.75	2.38	2.03	2.79
**Schizophrenia spectrum disorders**						
Yes	3.00	2.16	4.18	1.81	1.60	2.06
**Administrative health region**						
1 – Erie St. Clair	0.64	0.36	1.14	0.52	0.41	0.65
2 – South West	0.69	0.43	1.12	0.72	0.61	0.86
3 – Waterloo Wellington	0.61	0.39	0.97	0.53	0.44	0.63
4 – Hamilton Niagara Haldimand Brant	0.82	0.56	1.19	0.57	0.49	0.67
5 – Central West	0.30	0.16	0.54	0.50	0.41	0.61
6 – Mississauga Halton	1.18	0.81	1.71	0.74	0.63	0.86
8 – Central	0.45	0.30	0.68	0.61	0.53	0.71

© 2023 de Oliveira C et al. *JAMA Network Open*.

9 – Central East	0.82	0.57	1.18	0.76	0.66	0.87
10 – South East	0.71	0.43	1.18	0.70	0.58	0.86
11 – Champlain	1.20	0.85	1.69	0.79	0.68	0.91
12 – North Simcoe Muskoka	0.38	0.21	0.70	0.50	0.40	0.62
13 – North East	0.55	0.25	1.22	0.53	0.41	0.69
14 – North West	0.57	0.22	1.48	0.40	0.28	0.57
**Year**						
2013	1.15	1.13	1.17	1.09	1.08	1.10
2014	1.39	1.35	1.43	1.23	1.21	1.25
2015	1.69	1.62	1.76	1.39	1.36	1.43
2016	2.16	2.05	2.28	1.59	1.54	1.64
2017	2.76	2.59	2.95	1.77	1.70	1.84
2018	3.55	3.28	3.84	1.93	1.85	2.02
2019	4.56	4.15	5.01	2.06	1.96	2.17

* this is a very small, non-zero value

Legend: CI – confidence interval; ADHD – attention deficit hyperactivity disorder

Note: the reference case for each variable is as follows – sex: female; age group: 0-3; migrant status: long-term resident; neighbourhood income quintile: 1 – low; rural residence: no; chronic physical, behavioural and mental health conditions: no (i.e., absence of condition), administrative health region: 7 – Toronto Central; year: 2012.

Source: Administrative health care data housed at ICES, Toronto Ontario

© 2023 de Oliveira C et al. *JAMA Network Open*.
