# Supplementary material for: Mental Health Care Use Among Children and Adolescents With High Health Care Costs in Ontario, Canada
Source: JAMA Netw Open. 2023 May 12;6(5):e2313172. doi: 10.1001/jamanetworkopen.2023.13172 (PMC10182426; doi:10.1001/jamanetworkopen.2023.13172)
Supplement: Supplement 2. — Data Sharing Statement [file jamanetwopen-e2313172-s002.pdf]

Data Sharing Statement

de Oliveira. Mental Health Care Use Among Children and Adolescents With High Health Care Costs in Ontario, Canada. *JAMA Netw Open*. Published May 12, 2023. doi:10.1001/jamanetworkopen.2023.13172

Data
Data available: No
